# 非小细胞肺癌免疫治疗生物标志物研究进展

**DOI:** 10.3779/j.issn.1009-3419.2021.102.55

**Published:** 2022-01-20

**Authors:** 昌 江, 玲 易, 翔 高, 洪涛 张, 树才 张

**Affiliations:** 1 101149 北京，首都医科大学附属北京胸科医院北京市结核病胸部肿瘤研究所肿瘤内科 Department of Medical Oncology, Beijing Chest Hospital, Capital Medical University, Beijing Tuberculosis and Thoracic Tumor Research Institute, Beijing 101149, China; 2 101149 北京，首都医科大学附属北京胸科医院北京市结核病胸部肿瘤研究所中心实验室 Department of Laboratory, Beijing Chest Hospital, Capital Medical University, Beijing Tuberculosis and Thoracic Tumor Research Institute, Beijing 101149, China

**Keywords:** 非小细胞肺癌, 免疫治疗, 生物标志物, PD-L1, TMB, NSCLC, Immunotherapy, Biomarker, PD-L1, TMB

## Abstract

肺癌是最常见的恶性肿瘤之一，其发病率和死亡率位居所有恶性肿瘤首位。近年来，随着肿瘤免疫学的迅速发展，以细胞毒性T淋巴细胞相关蛋白4（cytotoxic T lymphocyte-associated antigen-4, CTLA-4）、程序性细胞死亡蛋白1（programmed cell death-1, PD-1）/程序性细胞死亡配体1（programmed cell death ligand 1, PD-L1）为靶点的免疫检查点抑制剂（immune checkpoint inhibitors, ICIs）不断进入临床实践，标志着免疫治疗的重大突破。免疫治疗的出现彻底改变了非小细胞肺癌（non-small cell lung cancer, NSCLC）的治疗现状，但仅有少部分患者能从中持久性获益。因此，如何筛选免疫治疗的获益人群，进一步提高免疫治疗的疗效是当下研究热点。本文聚焦NSCLC免疫治疗相关的生物标记物，并对近年NSCLC免疫治疗生物标志物研究现状和进展进行综述，为免疫治疗的临床实践提供参考。

## Abstract (en)

Lung cancer is one of the most prevalent malignancies with the highest morbidity and mortality rates worldwide. In recent years, with the development of immune-oncology research and several therapeutic antibodies have reach the clinic, many breakthroughs have been made in immunotherapy. The advent of immunotherapy has revolutionized the treatment of NSCLC, but the response and durable clinical benefit are only observed in a small subset of patients. Therefore, strategies to screen the potential beneficial population and improve the efficacy of immunotherapy remain an essential topic. In the current article, the author review the biomarkers that have potential to better predict responders to immunotherapy and to provide ideas for the clinical application of immunotherapy.

非小细胞肺癌（non-small cell lung cancer, NSCLC）是全球最常见的恶性肿瘤之一，其死亡率也居恶性肿瘤首位。既往治疗主要是以铂类为基础的双药化疗，但传统化疗不良反应多且疗效有限。免疫治疗开启了肿瘤治疗的新时代，为肿瘤的治疗模式带来了革命性改变。尽管免疫治疗已经取得突破性进展，但仅有一部分患者产生应答并从中持久性获益。在NSCLC中，不区分治疗人群，免疫治疗的客观缓解率（objective response rate, ORR）在20%左右^[1, 2]^，即使在程序性细胞死亡配体1（programmed cell death ligand 1, PD-L1）表达大于50%的人群中，ORR也不到50%^[3, 4]^。因此，为了更好地提高免疫治疗疗效，达到精准治疗的目的，肿瘤免疫治疗生物标志物的研究和探索备受重视。本文聚焦NSCLC免疫治疗相关的生物标记物，并对近年NSCLC免疫治疗生物标志物研究现状和进展进行综述，以便对其有更加深入、更加全面的认识，为免疫治疗的临床实践提供参考。

## 1 PD-L1表达

PD-L1是一种免疫检查点分子，与T细胞表面的程序性细胞死亡蛋白1（programmed cell death-1, PD-1）结合后可以抑制T细胞的激活，使肿瘤细胞发生免疫逃逸，促进肿瘤发展^[5]^。多项临床研究^[4, 6, 7]^表明，PD-L1的表达与NSCLC患者的ICIs治疗结局相关。Keynote-024和Keynote-042研究均为多中心、Ⅲ期随机对照试验，旨在比较Pembrolizumab与含铂化疗在无表皮生长因子受体（epidermal growth factor receptor, *EGFR*）、间变性淋巴瘤激酶（anaplastic lymphoma kinase, *ALK*）驱动基因突变的初治NSCLC中的疗效。Keynote-024研究^[4, 8]^将PD-L1肿瘤细胞阳性比例分数（tumor proportion score, TPS）≥50%患者纳入研究，与含铂化疗组相比，Pembrolizumab组具有更高的ORR（44.8% *vs* 27.8%）、更长的无进展生存期（progression-free survival, PFS）（10.3个月*vs* 6.0个月；HR=0.50，95%CI：0.37-0.68）和总生存期（overall survival, OS）（30.0个月*vs* 14.2个月；HR=0.63，95%CI：0.47-0.86）。2020年欧洲肿瘤内科学会（European Society for Medical Oncology, ESMO）年会上，Keynote-024研究^[9]^5年OS数据进行了更新，相较于化疗组，Pembrolizumab组具有更长的OS（26.3个月*vs* 13.4个月），5年OS率也显著高于化疗组（31.9% *vs* 16.3%）。Keynote-024试验的结果也使得美国食品药品监督管理局（Food and Drug Administration, FDA）批准Pembrolizumab作为驱动基因突变阴性且PD-L1 TPS≥50%的晚期NSCLC一线治疗方案。Keynote-042^[3]^将纳入研究人群拓展至PD-L1 TPS≥1%，研究结果显示Pembrolizumab组比化疗组具有更长OS（16.7个月*vs* 12.1个月；HR=0.81，95%CI：0.71-0.93）；亚组分析发现PD-L1 TPS≥50%组OS获益显著（20个月*vs* 12.2个月；HR=0.69，95%CI：0.56-0.85）；TPS 1%-49%亚组中，Pembrolizumab组OS未见明显获益。Pembrolizumab组内PD-L1 TPS≥50%的患者占比较高（299/637）及该群体较好的OS获益可能是整体分析中Pembrolizumab组OS获益的重要原因。Keynote-042研究进一步证实并巩固了Pembrolizumab在PD-L1高表达晚期NSCLC一线治疗地位；对于PD-L1 TPS 1%-49%的患者，Pembrolizumab单药治疗并不是一线治疗的最佳选择。

IMpower131将既往未经治疗的鳞状NSCLC患者按1:1:1的比例随机分配至Atezolizumab+卡铂/紫杉醇（A+CP）组、Atezolizumab+卡铂/白蛋白结合型紫杉醇（A+CnP）组、卡铂/白蛋白结合型紫杉醇（CnP）组。在PD-L1高表达亚组中，相较于CnP组，A+CnP组患者具有更长的PFS（10.1个月*vs* 5.1个月；HR=0.41，95%CI：0.25-0.68）和OS（23.4个月*vs* 10.2个月；HR=0.48，95%CI：0.29-0.81）。在PD-L1低表达和阴性亚组中A+CnP未见明显PFS和OS获益^[7]^。该研究数据表明，仅有PD-L1高表达亚组患者接受免疫联合化疗后PFS和OS可获益。

尽管PD-L1是FDA批准的首个用于ICI治疗的预测性生物标志物，其在临床应用中仍面临一些挑战。Checkmate017/057的研究结果，使Nivolumab被FDA批准用于NSCLC的二线治疗。与PD-L1 TPS≥1%的NSCLC患者可以从Pembrolizumab一线单药治疗获益不同，无论患者PD-L1表达与否，均能从Nivolumab二线单药治疗获益^[10]^。OAK研究^[11]^比较Atezolizumab与多西他赛在既往接受过治疗的NSCLC患者中的治疗效果，纳入患者不限病理类型和PD-L1表达状态。研究表明，无论PD-L1表达状态如何，Atezolizumab组均具有更长的OS。Keynote-189研究结果^[12]^显示，任何PD-L1表达状态下，非鳞NSCLC患者均能从Pembrolizumab联合化疗中获益。PD-L1表达对不同ICI、免疫单药或免疫联合化疗疗效预测具有不一致性，使其临床应用受到一定限制。除此之外，PD-L1的检测尚未标准化，有多种平台、多种抗体用于PD-L1检测，不同平台采用不同的评分系统评估PD-L1表达水平，且无严格的标准界定PD-L1的阴性和阳性^[13]^，而且，病理学家对PD-L1表达的评分在不同观察者之间的重复性较差^[14]^。免疫组化（immunohistochemistry, IHC）是评估肿瘤组织PD-L1表达最常用的方法，但PD-L1的表达是一个动态过程，受γ干扰素（interferon-γ, IFN-γ）、化疗和放疗等多种因素影响^[15]^，IHC不能有效反映该过程；同时，PD-L1的表达除了在肿瘤组织内存在异质性，不同转移部位表达也存在异质性，肾上腺、肝脏和淋巴结转移灶中表达最高，而骨和脑转移灶中表达较低。因此，IHC并不能反映肿瘤组织真实的PD-L1表达状态^[16]^，影响了PD-L1预测准确性。据报道，IHC的预测准确率 < 30%^[17]^。不同部位PD-L1表达水平的预测价值也不同，肺或远处转移灶中PD-L1的表达水平与临床获益呈正相关，而在淋巴结转移灶的PD-L1表达水平与临床获益无关^[18]^。

## 2 肿瘤突变负荷（tumor mutation burden, TMB）和肿瘤新抗原

### 2.1 TMB

TMB定义为肿瘤基因组去除胚系突变后的体细胞突变总数^[19, 20]^。这些基因编码区域的体细胞突变可以诱导新抗原的产生，肿瘤TMB越高，新抗原产生越多，肿瘤免疫原性越高，诱导的T细胞反应越强^[21]^。因此，高肿瘤TMB与ICIs临床应答存在显著相关性。CheckMate026是一项随机、开放标签的Ⅲ期临床研究，旨在比较Nivolumab与化疗在PD-L1表达阳性的初治NSCLC患者中的疗效^[22]^。在PD-L1≥5%的患者中，Nivolumab组与化疗组的PFS和OS无显著差异。探索性分析TMB对临床结局影响时发现，在高TMB患者（≥243 mut/Mb）中，Nivolumab组比化疗组具有更好的ORR（47% *vs* 28%）和更长的PFS（9.7个月*vs* 5.8个月），OS无差异，可能与化疗组内68%的高TMB患者交叉至Nivolumab组相关。CheckMate568是一项单臂Ⅱ期临床研究，该研究结果表明，晚期NSCLC患者接受Nivolumab联合Ipilimumab双免疫治疗的ORR与TMB呈正相关，TMB≥10 mut/Mb的患者ORR和PFS明显优于TMB < 10 mut/Mb的患者（44% *vs* 12%；7.1个月*vs* 2.6个月），且与PD-L1表达水平无关^[23]^。在此基础上，Checkmate227研究中以TMB≥10 mut/Mb作为高TMB的界值，在PD-L1≥1%的高TMB的晚期NSCLC患者中，Nivolumab联合Ipilimumab双免疫治疗的ORR和PFS均明显优于化疗（45.3% *vs* 26.9%；7.2个月*vs* 5.4个月；HR=0.58，95%CI：0.41-0.81；*P* < 0.001）^[24]^。在PD-L1 < 1%的高TMB患者中，也能看到相似结果。但2年OS探索性分析显示接受Nivolumab联合Ipilimumab治疗的患者中，高TMB和低TMB人群OS获益无显著差异^[25]^。另一项研究^[26]^表明，接受抗PD-1/PD-L1治疗的NSCLC中，TMB≥20 mut/Mb的患者OS显著延长。Huang等^[27]^设计了一项回顾性研究，在真实世界中评估TMB与NSCLC患者免疫治疗临床获益的关系，其结果表明，高TMB（≥10 mut/Mb）患者的临床获益明显优于低TMB患者。除了组织中的TMB，血液TMB（blood TMB, bTMB）也被证实具有预测免疫治疗疗效的价值。Gandara等^[28]^研究表明，在bTMB≥16 mut/Mb的晚期NSCLC患者中，接受Atezolizumab免疫治疗的PFS明显优于多西他赛化疗。MYSTIC研究的探索性分析结果^[29]^显示，在bTMB≥20 mut/Mb的晚期NSCLC患者中，Durvalumab联合Tremelimumab治疗比化疗具有更长的OS（21.9个月*vs* 10.0个月；HR=0.49，95%CI：0.32-0.74）和更高的12个月PFS率（38.6% *vs* 2.3%）。TMB作为免疫治疗的预测生物标志物，可以帮助筛选出更有可能从免疫治疗获益的人群，但仍然存在一定的不足。TMB在不同临床试验中的临界值不同，TMB检测平台不统一，检测结果的一致性存疑，TMB与ICIs临床疗效并非完全相关，因此TMB和bTMB作为免疫治疗标志物仍需进一步探索。

### 2.2 肿瘤新抗原

肿瘤新抗原是能被新抗原特异性T细胞受体识别的外来蛋白，因其可以引起T细胞反应，被认为与ICIs应答相关^[30, 31]^。一项回顾性分析^[30]^表明，高的克隆性新抗原负荷与原发性肺腺癌较长的OS相关，除此之外，高克隆性新抗原负荷与更好的抗PD-1应答相关。Rizvi等^[32]^研究结果也表明，新抗原负荷高的NSCLC患者具有更好的临床获益。通过预测突变肽和野生肽与主要组织相容性复合体-1（major histocompatibility complex class 1, MHC-1）亲和力间的差异，进而判断肿瘤新抗原的免疫原性，称为差异相似性指数（differential agretopicity index, DAI）^[33]^。DAI值高表明突变肽与MHC的亲和力高于野生肽，可以诱导产生更多的免疫应答。相较于TMB和传统的新抗原负荷，DAI对于免疫治疗具有更好的预测效能^[34]^。

## 3 T细胞炎性基因表达谱（gene expression profile, GEP）

GEP是与抗原呈递、趋化因子表达、细胞毒活性等相关的基因表达谱，可以更加全面地反映肿瘤微环境的免疫状况^[35]^。GEP与Pembrolizumab疗效间的关系是Keynote-028研究的探索性终点之一，结果表明，高T细胞炎性GEP患者具有更好的临床获益，当TMB联合GEP或PD-L1可以更好地预测免疫获益人群^[36]^。POPLAR研究^[37]^的探索性分析显示接受Atezolizumab治疗的患者中，效应T细胞相关基因和IFN-γ相关基因高表达的群体具有更长的OS。

## 4 基因突变相关标志物

分子靶向治疗是*EGFR*或*ALK*敏感突变患者的标准治疗。尽管口服EGFR酪氨酸激酶抑制剂（tyrosine kinase inhibitor, TKI）可使患者明显获益，但最终仍因不可耐受的毒副作用或进展停止治疗。既往的试验^[38, 39]^表明，二线单药免疫治疗在*EGFR*/*ALK*突变亚组无明显生存获益。Toki等^[40]^研究表明，*EGFR*突变样本中PD-L1表达低，即使肿瘤微环境中存在淋巴细胞，大部分也为无活性TIL。*EGFR*/*ALK*突变患者还具有较低的TMB。低TMB、PD-L1及CD8^+^ TIL可能是*EGFR*/*ALK*突变患者对免疫治疗应答低下的原因^[41, 42]^。IMpower150研究^[43]^是一项随机、开放标签设计的3期临床试验，在EGFR敏感突变亚组中，与BCP（贝伐珠单抗+卡铂+紫杉醇）相比，Atezolizumab+BCP改善了OS。因此，针对*EGFR*/*ALK*突变的NSCLC患者，在分子靶向治疗失败后是否使用免疫治疗，以及免疫治疗与其他治疗方式间如何组合都需要更深的研究。

*KRAS*是最常见的致癌因子之一，在肺腺癌中，*KRAS*常与抑癌基因*TP53*或*SKT11*发生共突变。*KRAS*或*TP53*突变的NSCLC表达更高水平PD-L1，可以从抗PD-1治疗中获益，*KRAS*/*TP53*共突变的患者获益更为显著^[44]^。*KRAS*基因突变存在多种亚型，各突变亚型与免疫治疗相关性并不一致。癌症基因图谱（the Cancer Genome Atlas, TCGA）数据库分析显示，*KRAS* G12D/TP53共突变组PD-L1表达水平和免疫细胞浸润水平明显降低，因此，*KRAS* G12D/TP53共突变可能是肺腺癌患者抗PD-1/PD-L1免疫检查点抑制剂的阴性预测标志物^[45]^。

SKT11在细胞代谢、细胞生长和调节细胞极性等方面发挥重要作用。*SKT11*突变与“冷”肿瘤免疫微环境形成相关，*SKT11*/*LKB1*突变的肿瘤组织内细胞毒性CD8^+^ T淋巴细胞浸润减少^[46]^。在接受ICIs治疗的*KRAS*突变肺腺癌患者中，相较于SKT11野生型，*SKT11*突变患者PFS和OS更短，动物实验进一步证实SKT11缺失促进PD-1/PD-L1抑制剂耐药^[47]^。Ricciuti等^[48]^的研究也表明*SKT11*突变与*KRAS*突变肺腺癌患者较差的免疫治疗结果相关。因此，*SKT11*突变可能是免疫治疗阴性预测标志物。

*KEAP1*也是一种抑癌基因，既往的研究^[49, 50]^表明*KEAP1*基因的改变会引起氧化应激通路的失调，从而导肿瘤耐药和放疗抵抗。Chen等^[51]^分析了泛癌种中*KEAP1*突变与免疫治疗结局的关系，*KEAP1*突变患者OS明显缩短，尽管*KEAP1*突变与高TMB相关，但也引起肿瘤内CD8^+^ T淋巴细胞浸润较少，提示其与“冷”肿瘤免疫微环境的形成相关。另一项真实世界研究^[52]^也表明KEAP1突变是免疫治疗预后不良标志物。Ricciuti等^[48]^研究也显示*KEAP1*突变与*KRAS*突变的肺腺癌患者较差的免疫治疗结果相关。

DNA损伤应答修复（DNA damage response and repair, DDR）基因在DNA损伤修复过程中发挥重要作用。DDR改变造成基因组稳定性降低，增加肿瘤TMB，增强了肿瘤免疫原性^[41]^。研究^[53]^显示，接受免疫治疗的NSCLC中，DDR突变患者具有更好的ORR以及更长的PFS和OS。表明DDR突变预示更好的免疫治疗疗效。

*POLE*基因编码的DNA聚合酶ε和*POLD1*基因编码的DNA聚合酶δ1对DNA复制的校正和保真至关重要。*POLE*和*POLD1*基因突变可引起DNA损伤修复功能缺陷，造成更多基因突变的发生^[54]^。一项泛癌种研究^[55]^发现，*POLE*/*POLD1*突变与更高的TMB相关，接受免疫治疗的*POLE*/*POLD1*突变患者也具有更长的OS。因此，*POLE*/*POLD1*突变是免疫治疗潜在预测生物标志物。

超进展（hyperprogressive disease, HPD）是指患者接受免疫治疗后出现肿瘤加速生长的现象，常伴有生存质量下降和较差的预后。*MDM2*/*MDM4*扩增、*EGFR*扩增、11号染色体上*CCND1*、*FGF3*、*FGF4*、*FGF19*扩增可以加速肿瘤生长，与HPD的发生存在明显相关性^[56, 57]^。因此，上述基因突变存在的情况下，是否选用免疫治疗需更加谨慎。

## 5 外周血生物标志物

外周血抽取容易，侵入性低，使得外周血生物标志物极具吸引力。中性粒细胞淋巴细胞比（neutrophilto-lymphocyte ratio, NLR）是指中性粒细胞绝对数与淋巴细胞绝对数的比值，在全血细胞计数后即可获取，目前已引起极大的关注。高中性粒细胞浸润但低淋巴细胞浸润的肿瘤微环境可促进血管生成、抑制细胞凋亡，从而促进肿瘤的发展。在接受免疫治疗的晚期NSCLC患者中，基线高NLR是一个不良预后因素^[58-60]^。除了与预后相关外，NLR还可以作为一种预测标志物，对二线使用Nivolumab的NSCLC患者进行回顾性分析发现，NLR升高的患者治疗失败时间更短^[61]^。因此，NLR可以用于预测免疫治疗反应。除了NLR，血小板淋巴细胞比（platelet-to-lymphocyte ratio, PLR）、相对嗜酸性粒细胞计数（relative eosinophil count, REC）、相对淋巴细胞计数（relative lymphocyte count, RLC）、LDH等都被认为与免疫治疗疗效相关^[62]^。

可溶性PD-L1（solubale PD-L1, sPD-L1）是PD-L1的一种剪切变异体，其没有跨膜结构域。肿瘤患者血浆中sPD-L1高于健康人。sPD-L1可以诱导免疫抑制，sPD-L1的存在介导PD-L1抑制剂耐药^[63]^。接受免疫治疗的患者中，高sPD-L1亚组PFS和OS明显比低sPD-L1亚组短，多因素分析提示高sPD-L1是独立的预后不良因素^[64, 65]^。sPD-L1可作为免疫治疗疗效预测和预后的标志。

## 6 肠道微生物

人体肠道中存在的细菌、病毒、真菌等微生物共同构成了肠道菌群，肠道菌群大多数与人体呈共生关系，与机体相互作用，维持着肠道内的稳态，在机体免疫反应中发挥重要作用^[66]^。近年来研究表明肠道菌群组成可以影响免疫治疗的疗效。Katayama等^[67]^研究表明，接受ICI治疗的NSCLC患者肠道内乳酸菌属和梭菌丰度越高，治疗失败时间（time to failure, TTF）越长，对ICI治疗产生应答的患者肠道菌群种类更加多样。Jin等^[68]^对接受抗PD-1治疗的中国晚期NSCLC患者肠道菌群与临床结局的关系展开研究，结果显示肠道微生物群的多样性与抗PD-1治疗良好反应具有明显相关性，长双歧杆菌、普氏菌和*Alistipes putredinis*富集的患者具有更长的PFS。Routy等^[69]^研究显示，免疫治疗前肠道内*Akkermansia muciniphila*数量多的患者对免疫治疗具有更好的应答，在接受免疫治疗前或治疗期间使用过抗生素的患者PFS明显缩短。小鼠模型中，对ICIs有反应的肿瘤患者的粪便微生物移植到无菌或抗生素治疗的小鼠中，可以改善PD-1抑制剂的抗肿瘤效果。而对ICIs无反应患者的粪便微生物移植则无该效应，口服补充*Akkermansia muciniphila*使其再定植后，可以恢复PD-1抑制剂的抗肿瘤疗效。肠道菌群的多样性与菌种差异使肺癌患者对免疫治疗具有不同反应，这可能与肠道菌群影响免疫细胞功能、诱导免疫细胞浸润相关。

## 7 总结

免疫治疗的出现，进一步丰富了肿瘤治疗手段，也开启了晚期NSCLC治疗的新模式。虽然围绕免疫治疗生物标志物进行了许多研究，但仍面临诸多挑战。例如，虽然PD-L1和TMB与免疫治疗疗效存在相关性，但并非所有PD-L1高表达或高TMB患者对免疫治疗应答，而一些PD-L1低表达、不表达或低TMB患者仍能从免疫治疗中获益。单一生物标志物都存在不同程度的不足，会影响免疫治疗获益人群筛选的准确性。因此，通过多种生物标志物联合使用，构建稳定有效的免疫治疗预测模型并寻找更为有效的治疗模式，使接受免疫治疗的患者临床获益最大化，促进肿瘤免疫治疗向精准治疗方向迈进。
